# Seasonal Consumption of Cherries from Different Origins Affects Metabolic Markers and Gene Expression of Lipogenic Enzymes in Rat Liver: A Preliminary Study

**DOI:** 10.3390/nu13103643

**Published:** 2021-10-18

**Authors:** Ma. Josefina Ruiz de Azua, Álvaro Cruz-Carrión, Begoña Muguerza, Anna Arola-Arnal, Manuel Suarez

**Affiliations:** Nutrigenomics Research Group, Departament de Bioquímica i Biotecnologia, Universitat Rovira i Virgili, 43007 Tarragona, Spain; mariajosefina.ruiz@urv.cat (M.J.R.d.A.); alvarojavier.cruz@urv.cat (Á.C.-C.); begona.muguerza@urv.cat (B.M.); anna.arola@urv.cat (A.A.-A.)

**Keywords:** xenohormesis, polyphenols, photoperiods, seasonality, liver lipogenic enzymes

## Abstract

The phytochemical composition of fruits, especially polyphenols, depends on the environmental conditions under which these fruits are cultivated and the agronomic practices followed. Therefore, the consumption of fruits from different origins, with different polyphenol signatures, could have differential effects on health. In addition, recent studies have shown that variation in the biological rhythms due to changes in the photoperiod in the different seasons differentially affect the metabolism in animal models, thus conditioning their response to food consumption. Considering all, this article evaluates the effects of consumption of sweet cherry from different sources, local (LC) and non-local (nLC), on plasma metabolic parameters and the gene expression of key enzymes of lipid metabolism in Fischer 344 rats under photoperiods simulating different seasons. Animals were classified into three photoperiods (L6, L12 and L18) and three treatments (LC, nLC and VH). Both the photoperiod and the treatments significantly affected the evaluated parameters. An effect of the photoperiod on triacylglycerides, non-esterified fatty acids and the mRNA concentration of crucial enzymes from the hepatic lipid metabolism was observed. Furthermore, the consumption of fruit in L12 lowered blood glucose, while the different treatments affected the hepatic expression of genes related with lipidic enzymes.

## 1. Introduction

Polyphenols are secondary metabolites synthesized by plants, which, thanks to their characteristic phenolic structure, have certain protective effects against chronic diseases, such as cardiovascular [1,2], neurodegenerative [3] and metabolic pathologies [4,5], as well as activity against some types of cancer [6,7,8]. These phytochemicals are present in fruits, vegetables, tea, chocolate, coffee and oils, among others. Therefore, a regular consumption of these foods would provide health benefits [1,9]. Cherries, for their part, have a low caloric content and not only provide sugars, fiber, vitamin C, potassium, tryptophan, serotonin and melatonin, but also guarantee a significant contribution of bioactive phytochemicals [10]. Although the concentration and composition of these phytocompounds depend on various factors, both sweet cherries (*Prunus avium L.*) and sour cherries (*Prunus cerasus L.*) are rich in polyphenols, mainly anthocyanins, hydroxycinnamic acid and flavonoids [11]. In particular, their consumption has been associated with anti-inflammatory, anti-oxidative and antifungal properties, in both animal and human models [12,13,14].

Further, the day/night rhythm, as well as the wake/sleep rhythm, regulates many physiological processes in mammals, including body temperature, hormonal secretion, food intake and lipid metabolism. Further seasonal changes and the duration of daylight exposure modulate the circadian rhythm, thus influencing the clock genes and resulting in direct and indirect effects on metabolic parameters. Consequently, changes in the photoperiod can produce alterations that predispose to obesity and increase the risk of metabolic syndrome [15].

Hormesis is the concept that defines the process by which living beings adapt to low-dose exposure to external harmful stimuli to achieve resistance to future exposures and thus enhance the survival of the species. The development of signaling pathways including transcription and regulatory factors in the gene expression of cytoprotective proteins are some of these adaptive responses [16]. For example, plants develop a distinctive phytochemical composition, primarily polyphenols, that depends on the environment and the stressful stimuli that they receive [17]. Within this context, xenohormesis is defined as inter-species hormesis, where organisms are able to detect these signaling molecules synthesized by other species and thus adapt and adequately prepare themselves for adverse situations [18]. In this sense, the consumption of fruits and vegetables (and their phytochemicals) by heterotrophs would serve as an alert signal, allowing their adaptation, stimulation and cellular strengthening in the face of adversity and stress [17]. However, recently, it has been postulated [19] that out-of-season consumption could introduce misleading signals, thus altering the metabolism.

For example, an increase in the gene expression of enzymes related to the transport and b-oxidation of fatty acids was observed in the soleus and gastrocnemius muscles of normal weight rats that ingested cherry during the off-season, while in obese rats their consumption produced an increase in the glycemia and insulinemia, accompanied by a better use of lipids [20]. Furthermore, it has been observed that the antioxidant effect of cherries from different origins depend on the photoperiod of consumption, mainly preventing oxidative stress when they are ingested in the season of consumption [21]. This differential effect could be attributed to differences in the post-harvest treatments, transport and storage conditions, including time, temperature and exposure to light, which significantly affect the polyphenol content [22]. Therefore, it could be thought that the differential effect of fruit consumption depends not only on the photoperiod to which the animals are exposed, but also on the origin of the fruit, which would cause a characteristic polyphenolic composition and that it can be modified by various post-harvest factors.

For all the aforementioned, the objective of the present study was to investigate the consequences of cherry consumption from different sources, both local and non-local, by male Fischer 344 rats exposed to different photoperiods on metabolic parameters.

## 2. Materials and Methods

### 2.1. Fruits: Preparation and Characterization

For the experiment, Brooks sweet cherries that were cultivated and harvested in different countries, Spain (LC: local cherry) and Chile (nLC: non-local cherry), were used. After discarding the pips, they were frozen in liquid nitrogen, ground and lyophilized at −55 °C using the Telstar LyoQuest lyophilizer (Thermo Fisher Scientific, Barcelona, Spain). The different lyophilized fruits presented the following nutritional composition expressed as a function of their dry weight (dw). On the one hand, LC contained 64.1 mg/g of protein, 10.1 mg/g of total lipids, 795 mg/g of carbohydrates, of which 433 mg/g was sugars and 109.5 mg/g was dietary fiber. On the other hand, nLC had 55.1 mg/g of protein, 7 mg/g of total lipids and 807.8 mg/g of carbohydrates, of which 500 mg/g was sugars and 107.4 mg/g was dietary fiber [21]. The total content of polyphenols (TPC), anthocyanins (TAC), flavanol (TFaC) and flavonols (TFoC) in the LC and nLC extracts were quantified according to the procedures described by Iglesias-Carres [23,24]. Such compounds were expressed in mg equivalents of gallic acid (GAE), cyanidin-3-*O*-rutinoside (Cy3R), catechin (Cat), Quercetin (Quer), respectively. The LC extracts presented 8.17 ± 0.20 (mg GAE/g dw), 1,31 ± 0.02 (mg Cy3R eq/g dw), 0.44 ± 0.002 (mg Cat eq/g dw), 0.55 ± 0.00 (mg Quer eq/g dw) and the nLC 7.64 ± 0.41 (mg GAE/g dw), 1.23 ± 0.03 (mgCy3R eq/g dw), 0.38 ± 0.03 (mg Cat eq/g dw) and 0.63 ± 0.05 (mg Quer eq/g dw) [21].

### 2.2. Animals

The Animal Ethics Committee of the University Rovira i Virgili (Tarragona, Spain) approved all of the procedures (Project identification code: 9495; file number: FUE-2017-00499873). Seventy-two 8-week-old Fischer 344 (F344) rats were acclimatized for 4 days. (Charles River Laboratories, Barcelona, Spain). We used F344 rats due to their high sensitivity to the amount of daily light hours, having consequences on metabolic, physiological and behavioral parameters [25,26,27]. The animals were housed in pairs in cages at 22 °C and, to simulate the differences in the length of the day in the different seasons of the year, were divided into three groups: short-day photoperiod (*n* = 24, L6, 6 h of light and 18 h of darkness); standard photoperiod (*n* = 24, L12, 12 h of light and 12 h of darkness); long-day photoperiod (*n* = 24, L18, 18 h of light and 6 h of darkness). Animals underwent 4 weeks of adaptation where they were fed *ad libitum* with a standard diet (STD) (Panlab A04, Barcelona, Spain). After acclimatization, within each photoperiod, animals were divided into 3 groups according to the treatment: vehicle (VH, *n* = 8), LC (*n* = 8) or nLC (*n* = 8). The VH-treated group was supplemented with a sugar solution (glucose: fructose 1:1, 21.2 mg per kg of body weight), to equal the amount of carbohydrates that were administered to the animals treated with the fruits. It should be noted that, considering the weight of the rats and the amount of food consumed, the contribution of the vehicle only represented a contribution of 0.063 kcal/day, which would be equivalent to 0.11% of their daily energy intake [21]. Therefore, the metabolic impact of this sugar load was not relevant in the context of the whole diet. Treated animals were administered a dose of 100 mg of freeze-dried fruit per kg body weight (diluted in water) one hour after the lights were switched on. The 3 treatments were administered by voluntary licking through a syringe to guarantee the consumption of the entire dose. After 7 weeks of treatment, the animals were sacrificed by decapitation, having their last dose one hour before being guillotined. The blood was collected from the neck, in non-heparinized tubes, incubated for 1 h at room temperature and immediately centrifuged at 1200× *g* for 15 min to collect the serum. The liver and serum obtained were stored at −80 °C.

### 2.3. Serum Analysis

Circulating levels of glucose, triacylglycerides (TAG), total cholesterol (TC), HDL cholesterol (HDL-c), LDL cholesterol (LDL-c) (QCA, Amposta, Spain) and non-esterified fatty acids (NEFAs) (WAKO, Neuss, Germany) were determined by enzymatic colorimetry. Serum insulin levels were determined using a rat insulin ELISA kit (Millipore, Barcelona, Spain). The homeostatic model assessment (HOMA) index was calculated, using the equation (Glycemia (mmol/L) * Insulinemia (mU/dL)/22.5).

### 2.4. Cardiovascular Risk Indices

Although, traditionally, the quantification of LDL-c, HDL-c and TC is used to diagnose cardiovascular diseases and disorders, today there is evidence that their values, taken separately, are not as effective as the relationships between them. Consequently, different ratios are used to assess the existence of serum lipid alteration [28,29,30,31]. Therefore, the following relationships were calculated in the present study: atherogenic index (AI: Log (TAG/HDL-c)), cardiovascular risk 1 (CR1: (TC/HDL-c)), cardiovascular risk 2 (CR2: (LDL-c/HDL-c)) and atherogenic coefficient (At.C: (TC-HDL-c)/HDL-c). For their calculation, the values of the biomarkers expressed in mmol/L were used, which were quantified as detailed in Section 2.3 “Serum Analysis”.

### 2.5. Hepatic Gene Expression Analysis 

Total liver RNA was extracted using TRIzol Reagent (Thermo Fisher Scientific, Illkirch-Graffenstaden, France) following the supplier’s instructions. cDNA was synthesized by reverse transcription using High-Capacity cDNA Reverse Transcription (Thermo Fisher Scientific, Illkirch-Graffenstaden, France). Specific cDNA amplification was performed by real-time polymerase chain reaction (RT-qPCR) using iTaq Universal SYBR Green Supermix (Bio-Rad, Barcelona, Spain). The primers used for the different genes were obtained from Biomers.net (Ulm, Germany) and are described in Supplementary Table S1. The genes of interest were those related to lipid metabolism: Carnitine palmitoyltransferase 1-α (Cpt1α), Acetyil-coenzyme A carboxylase (Acc1), fatty acid translocase homolog of CD36 (Cd36), fatty acid synthase (Fas1), sterol regulatory element-binding protein 1 (Srebp-1c), hydroxyacyl-CoA dehydrogenase (Had) and fatty acid transporter 5 (Fatp5). The relative expression for each gene was calculated as a percentage of the L18-VH group, considering that cherry season consumption is in summer. The Pfaffl [32] method was used taking into account the efficiency of each particular primer and using Ppia as the endogenous control gene.

### 2.6. Statistical Analysis 

Data are shown as mean ± SEM. The statistical analyses were carried out with the SPSS Statistics 22 software (SPSS Inc., Chicago, IL, USA). Outliers were discarded and normality was evaluated using the Shapiro–Wilk test and homogeneity with the Levene’s test. For those values that met these criteria, a two-way analysis of variance (ANOVA) was performed (Photoperiod x Treatment). In cases where there was a significant effect or trend of any of the variables, a one-way ANOVA was continued using a DMS post-hoc test to determine the differences between the different groups. The analysis by the Student’s *t*-test was used to compare pairs of groups within the same photoperiod or the same treatment in different photoperiods. Kruskal–Wallis and Mann–Whitney U tests were performed, as appropriate. The threshold of statistical significance was established at *p* < 0.05 and the trend one at 0.05 < *p* <1.0.

## 3. Results

Sweet cherry is a typical spring/summer fruit, whose consumption in season corresponds to long days (L18). It should be noted that, in our previous manuscript, carried out with the same group of animals, we did not observe significant differences neither in caloric consumption nor in the weight of the animals. Nevertheless, the animals exposed to L12 presented a higher amount of MWAT than those animals exposed to L6 or L18. However, the rats exposed to L6 had the lowest % of body fat and muscle compared to the rest of the photoperiods [21]. Therefore, this evidence would reflect the importance of the amount of light hours to which they are exposed on the body composition of animals, which is accompanied by changes in metabolic parameters observed in the present study. 

### 3.1. Exposure to Different Photoperiods Significantly Affected Triglycerides and Blood NEFAs

There was a significant effect of exposure to different photoperiods on plasma TAG levels (*p* = 0.03, two-way ANOVA) (Table 1). Although no differences were found between the VH of each photoperiod group, it was observed that, in L6, the animals treated with nLC presented significantly higher levels of TAG than those treated with LC (*p* = 0.01). In turn, consuming nLC in L6 also tended to increase TAG levels compared to VH (*p* = 0.06). Furthermore, nLC in different photoperiods had a differential effect, since, in L6, it was associated with higher plasma TAG levels than in L12 or L18 (*p* = 0.04; *p* = 0.006 respectively).

Moreover, NEFAs levels were also affected depending on the photoperiod (*p* = 0.01; two-way ANOVA), while exposure to different amounts of light along with the administration of different treatments tended to have a significant effect (*p* = 0.05; one-way ANOVA) (Table 1). Specifically, we observed a dramatic decrease in serum levels of NEFAs from those animals that consumed both types of cherries in the L18 photoperiod compared to their respective VH and also in relation to the other L6 and L12 photoperiods. A similar behavior was observed in the animals in group L6, although the differences between treatments were not statistically significant.

TC, HLD-c and LDL-c levels were not significantly affected by the photoperiod or by the consumption of any type of fruit (Table 1). However, it was observed that the animals treated with LC in L6 and L18 tended to present a higher concentration of LDL-c than the L12-LC group (*p* = 0.078, *p* = 0.066, respectively; one-way ANOVA). Nevertheless, a similar conduct was also observed in relation to HDL-c in these groups, where L18-LC animals tended to have a higher level than L12-LC ones (*p* = 0.055). Furthermore, cherry consumption in L18 seemed to have a beneficial effect on this biomarker, when higher levels in LC and nLC were observed than in their respective VH (*p* = 0.036, *p* = 0.062, respectively).

### 3.2. Exposure to L12 Increased Blood Glucose, While Cherry Consumption Normalized It 

As observed in Table 1, cherry consumption tends to have an effect on plasma glucose levels (*p* = 0.087; two-way ANOVA). Specifically, its intake, regardless of its origin, decreased glucose levels with respect to VH consumption in L12 (L12-LC vs. L12-VH *p* = 0.021; L12-nLC vs. L12-VH *p* = 0.046). However, in the other photoperiods this differential effect between fruit consumption and their respective VH was not observed. 

On the other hand, there is a differential effect between the VH of different photoperiods, since the animals belonging to the L12-VH group presented higher levels of blood glucose in relation to L18-VH ones (*p* = 0.04; Student’s *t*-test) (Table 1). 

### 3.3. Insulin Levels and HOMA Index Tended to Be Affected by Exposure to Different Photoperiods and Treatments, Concomitantly

By applying a two-way ANOVA, it could be seen that the interaction between exposure to different photoperiods and the treatment tended to affect plasma insulin levels in animals (*p* = 0.064) (Table 1). Specifically, the L6-VH group had lower insulin levels than VH ones exposed to L12 or L18 (*p* = 0.08; *p* = 0.03, respectively; Student’s *t*-test). In addition, in L6, the intake of any type of cherry increased the plasma insulin levels compared to its respective VH (L6-LC vs. L6-VH *p* = 0.01; L6-nLC vs. L6-VH *p* = 0.03; Student’s *t*-test). Further, it was observed that the intake of LC in L18 tended to decrease insulinemia compared to its consumption in L6 (*p* = 0.058; Student’s *t*-test). In relation to these results, the HOMA index is closely linked to the insulin and blood glucose values; therefore, a similar behavior could be observed. In this sense, the animals that were exposed to short days and that consumed any type of cherry, presented a higher HOMA index than VH ones (L6-LC vs. L6-VH *p* = 0.03; L6-nLC vs. L6-VH *p* = 0.05; one-way ANOVA). Furthermore, those groups treated with VH in L12 showed a higher HOMA index than L6-VH (*p* = 0.03), while, compared with those exposed to a long photoperiod, this difference did not reach statistical significance (*p* = 0.06; Student’s *t*-test).

### 3.4. The Cardiovascular Risk Indices Were Affected by Both Photoperiod and Treatment

Although neither the rat exposure to different photoperiods nor the treatment affected the content of TC, HDL-c and LDL-c, relevant effects on their derived indices, namely, the relationship CR1 and the At.C (Table 1), were observed. Specifically, the L12-LC group presented higher CR1 and At.C than nLC and VH animals within the same photoperiod (CR1, *p* = 0.01, *p* = 0.01 and At.C, *p* = 0.01, *p* = 0.01, respectively). Furthermore, L12-LC animals also had these same high rates as those groups that received the same type of fruit in L6 or L18 ((TC/HDL-c) and At.C, *p* = 0.02, *p* = 0.00, respectively). Furthermore, exposure to different hours of daylight also affected AI. Particularly, rats that consumed LC in the L12 photoperiod presented higher AI than L18-LC animals (*p* = 0.061). It should be noted that, in L18, that simulated the fruit season, the treated animals had lower AI than their respective VH, although these differences were not significant. Therefore, taking into consideration all the indices, a chronic exposure to more hours of light, added to cherry consumption in its season, seems to have decreased cardiovascular risk and lowered the atherogenic power. 

### 3.5. Gene Expression of Acc1 and Fas1 Lipogenic Enzymes Was Increased by Chronic Exposure to the L18 Photoperiod

**Figure 1:**
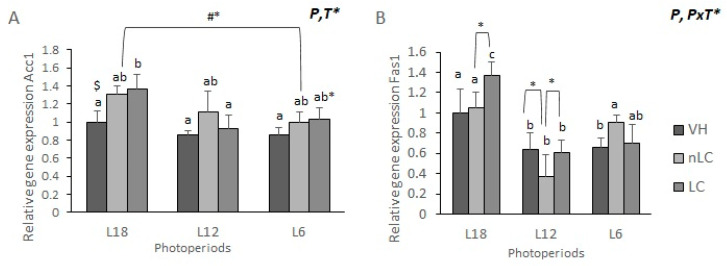
Gene expression of liver lipogenic enzymes. The mRNA levels of Acetyl-coenzyme A carboxylase (Acc1) (**A**) and fatty acid synthase (Fas1) (**B**) of male Fischer 344 rats treated for 7 weeks with vehicle (VH), non-Local cherries (nLC) or Local (LC), and exposed to different photoperiods (short; L6, standard; L12 or long; L18). Values expressed as mean ± SEM (*n* = 8). The values were normalized by the L18-VH group. P, photoperiod effect; T, treatment effect; P x T, photoperiod effect and treatment. (two-way ANOVA, *p* < 0.05). Different letters above the bars indicate significant differences (*p* < 0.05) (post-hoc DMS, one-way ANOVA). $ significant effect of treatment in the same photoperiod (Student’s *t*-test). * indicate trend (0.05 < *p* < 0.1). #* indicates trend (0.05> p > 0.1) with Student's t-test.

Due to the fact that photoperiod and treatment mainly influence the plasmatic lipid fraction, its impact on the gene expression of various hepatic enzymes involved in lipid oxidation and biosynthesis was evaluated to elucidate the involved metabolic pathways. mRNA levels are expressed as relative units, normalized with the L18-VH group. As it can be seen in Figure 1A, there was a significant effect of the photoperiod and a trend due to the treatment on the mRNA levels of the *Acc1* lipogenic enzyme (two-way ANOVA). Occasionally, animals treated with cherries and exposed to the L18 photoperiod tended to present higher levels of the *Acc1* than their control VH (L18-nLC *p* = 0.098; L18-LC *p* = 0.060; Student’s *t*-test). Furthermore, when comparing the effect of cherry consumption between different groups exposed to different hours of daily light, it can be seen that LC consumption in L12 was associated with a lower gene expression of this enzyme than in L18-LC animals (*p* = 0.031; one-way ANOVA). A similar effect occurred on the animals exposed to the L6 photoperiod, which tended to have lower mRNA levels relative to the L18 group, regardless of the origin of the ingested fruit. (L18-nLC vs. L6-nLC *p* = 0.051, Student’s *t*-test; L18-LC vs. L6-LC *p* = 0.092, one-way ANOVA). However, there were no significant differences between the VH groups.

On the other hand, exposure to different photoperiods significantly affected the levels of the hepatic *Fas1* mRNA (Figure 1B). Furthermore, their expression tended to be influenced by the interaction of photoperiod and treatment. Particularly remarkable, the group treated with nLC in L12 had lower mRNA levels than those who consumed the same fruit but in L18 or L6 (*p* = 0.00, *p* = 0.005, respectively; one-way ANOVA). In addition, the animals that were exposed to higher amount of light hours significantly increased the expression of this lipogenic enzyme, when comparing the different treatments with those of the L12 and L6 photoperiods. Specifically, the L18-VH group showed a higher mRNA concentration than L12-VH and L6-VH (*p* =0.038, *p* =0.047, respectively). Although no significant effect of the treatment alone was observed, it can be seen that, within the L18 group, the animals that received LC presented a higher expression of *Fas1* than their respective VH, while, compared with those that ingested nLC, this difference was not significant (*p* = 0.060). A similar effect occurred in the L12 photoperiod, where nLC consumption tended to decrease its expression with respect to LC (*p* = 0.096; Student’s *t*-test). However, when the animals were exposed to a few hours of light, a higher gene expression was observed when they received nLC than LC, despite not being significant.

### 3.6. The Gene Expression of Srebp-1c and Cpt1α Tended to Be Affected by Exposure to Different Photoperiods

**Figure 2:**
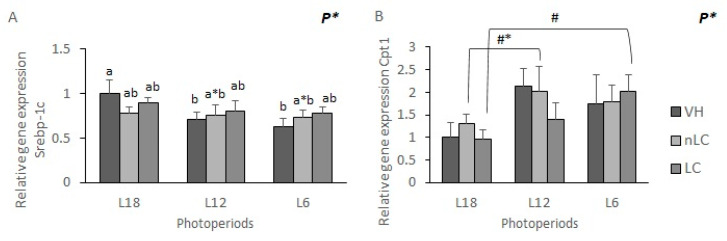
Gene expression of liver protein and enzyme. The mRNA levels of sterol regulatory element-binding protein 1 (srebp-1c) (**A**) and Carnitine palmitoyltransferase 1 α (cpt1α) (**B**), of male Fischer 344 rats treated for 7 weeks with vehicle (VH), non-Local cherries (nLC) or Local (LC), and exposed to different photoperiods (short; L6, standard; L12 or long, L18). Values expressed as mean ± SEM (*n* = 8). The values were normalized by the L18-VH group. P, photoperiod effect (two-way ANOVA, *p* < 0.05). Different letters above the bars indicate significant differences (*p* < 0.05) (post-hoc DMS, one-way ANOVA). # effect of the same treatment compared between different photoperiods (Student’s *t*-test). * indicate trend (0.05 < *p* < 0.1).

Exposure to different photoperiods tended to affect the mRNA levels of the *Srebp-1c* and *Cpt1α* enzyme in the different groups, when applying the two-way ANOVA analysis. On the one hand, as seen in Figure 2A, exposure to long days was associated with a higher expression of the *Srebp-1c* enzyme than on shorter days, both L12 and L6. (L18-VH vs. L12-VH *p* = 0.034; L18-VH vs. L18-6 *p* = 0.008). Regarding the expression of *Cpt1α*, it was observed that the VH exposed to L12 and L6 showed a higher expression than those exposed to L18, although these differences were not significant (*p* = 0.053, *p* = 0.368, respectively; Student’s *t*-test) (Figure 2B). Likewise, the animals that ingested LC and were exposed to short days showed a higher gene expression than those that consumed it on long days (*p* = 0.02; Student’s *t*-test).

### 3.7. The Gene Expression of Enzymes Related to Lipid Oxidation Was Not Significantly Affected by the Photoperiod or by the Treatment

**Figure 3:**
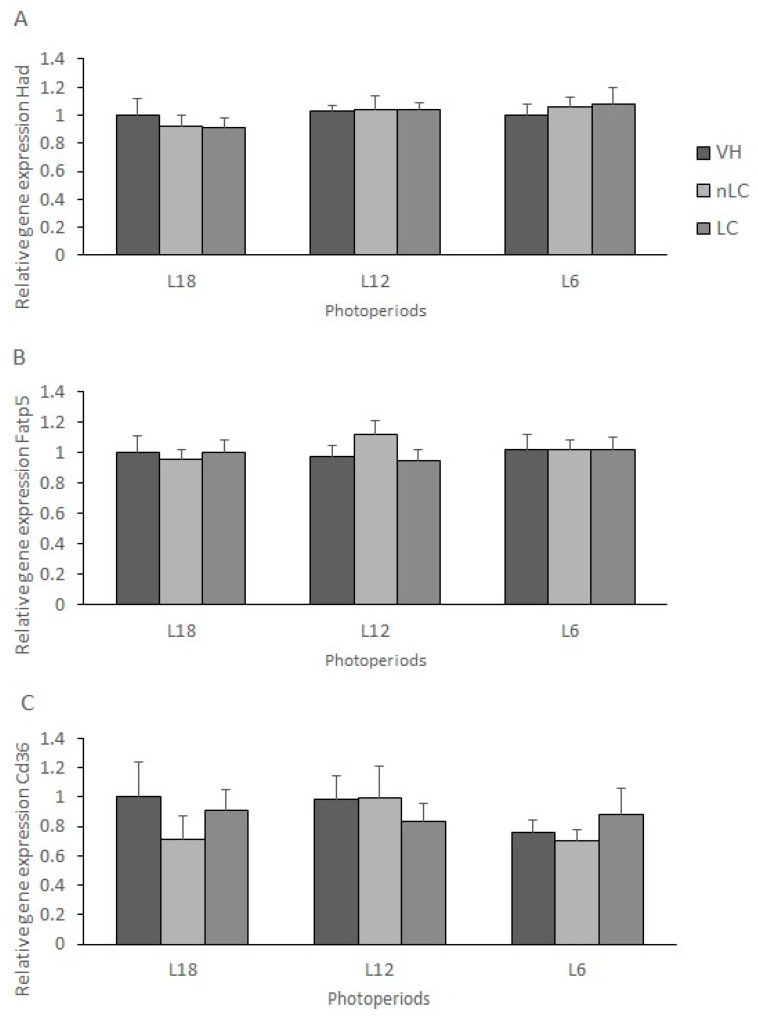
Gene expression of liver enzymes related to lipid metabolism. The mRNA levels of hydroxyacyl-CoA dehydrogenase (Had) (**A**), fatty acid transporter 5 (Fatp5) (**B**) and fatty acid translocase homolog of CD36 (Cd36) (**C**), of male Fischer 344 rats treated for 7 weeks with vehicle (VH), non-Local cherries (nLC) or Local (LC), and exposed to different photoperiods (short; L6, standard; L12 or long; L18). Values expressed as mean ± SEM (*n* = 8). The values were normalized by the L18-VH group.

In addition to understanding the metabolic pathways related to hepatic lipid biosynthesis, we also analyzed the expression of those enzymes that play a key role on fatty acid oxidation. On the one hand, neither the amount of daily light nor the different treatments affected the expression of *Had* and *Fatp5*, since no significant differences were observed among the groups (Figure 3A,B). However, in the mRNA concentration of the translocase *Cd36,* although it did not reach the significant difference, it seems that the consumption of cherry in L18 would decrease its expression compared to its respective VH (Figure 3C). Furthermore, it appears that exposure to short days would also decrease the expression of *Cd36* compared to long days, although, when cherry was consumed, the gene expression remained the same in both photoperiods.

## 4. Discussion

The development of the metabolic syndrome is multifactorial, where not only lifestyle and genetic predisposition have an influence, but environmental stimuli such as the number of hours of daylight to which individuals are exposed also affect the circadian rhythm and therefore to circulating biomarkers. In this sense, both the quantity and the quality of the food consumed play a fundamental role in the genesis of this disease [15,25,33]. Likewise, the polyphenols present in fruits and vegetables have a protective function against certain metabolic disorders [14]. 

In the present study, the animals received a daily dose of 100 mg freeze-dried fruit/kg body weight of sweet cherry. Considering the humidity of the fruit and applying the corresponding formula to translocate this dose to humans [34], this treatment is equivalent to a human equivalent dose of 6.46 g of fresh sweet cherry in a 70 kg person per day. The results demonstrate that the consumption of cherry in its natural season, L18, could stimulate the gene expression of lipogenic enzymes and a decrease in the serum concentration of NEFAs, at the same time that it improves insulin sensitivity. Consequently, we noted that the exposure to different photoperiods affected lipid metabolism, specifically the plasma concentration of TAG, NEFAs and the expression of lipogenic genes. Glycemia and insulinemia were influenced both by the photoperiod and by the consumption of cherries from different sources.

In relation to animals exposed to L18, it was observed that there were significant differences in the circulating levels of NEFAs. Specifically, those treated with VH presented higher levels of NEFAs, which would reflect a higher oxidation of TAG by peripheral tissues, than those treated with LC and nLC. Therefore, in the animals that consumed cherry, as it was expected, we observed the highest activity of the key enzymes for the *de novo* synthesis of hepatic fatty acids, since *Acc1* catalyzes the carboxylation of Acetyl-CoA to form malonyl-CoA, which serves as an inhibitor of mitochondrial b-oxidation and a precursor for the synthesis of Palmitoyl-CoA by *Fas1* [35]. In this sense, the increased gene expression of *Acc1* and Fas1 in season (L18-LC and L18-nLC), confirms the fact that its consumption could be stimulating lipogenesis. However, no significant differences were observed in the amounts of TAG between the groups of animals in L18, which could be due to a compensatory mechanism in the increase in gene expression of mitochondrial *Acc2* as a consequence of a decrease in or suppression of acc1 cytosolic [35]. It should be noted that, despite the fact that the TC levels were not modified by any of the treatments, the concentrations of LDL-c and HDL-c changed. Although the differences were not statistically significant, a beneficial effect was observed in animals that had consumed cherry in L18. Various studies demonstrate the inverse correlation between HDL-c concentration and cardiovascular events thanks to its role in the mobilization of cholesterol from peripheral cells to the liver, where it is catabolized and eliminated [36]. Furthermore, there is evidence on the cardioprotective properties of polyphenols, mainly anthocyanidins and their metabolites, because they increase the reverse transport of cholesterol and the concentration of HDL-c through the activation of the liver X receptor (LXR) and/or the regulation of lipid transporters [37,38,39]. Therefore, it was expected that, in the present study, the animals fed with cherry in L18 showed a higher average concentration of HDL-c, which reached even twice that observed in their respective VH. However, these beneficial effects were not seen in L6 or L12, demonstrating, once again, the importance of consuming the fruits in their natural season. Animals treated with VH also tended to have less LDL-c than those consuming LC and nLC. Although we did not analyze the gene expression of enzymes related to LDL-c and HDL-c metabolism, we can assume that cherry consumption in its season interferes with the activity of enzymes related to lipoprotein metabolism, either in its synthesis or in its excretion. For all the previously explained, we can understand that the consumption of cherry in its season, that is, L18, could be sending signals to decrease the use of fatty acids by peripheral tissues, at the same time that it stimulates the expression of lipogenic enzymes and the cholesterol synthesis by some kind of positive regulation.

On the other hand, no significant differences were observed in the levels of TAG, NEFAs or TC in the animals exposed to the L12 photoperiod. It can be seen that those animals that consumed nLC had higher HDL-c and LDL-c concentrations than the other treatments, but this change did not reach statistical significance. Our results highlight that, although the L12-nLC group had the highest HDL-c levels, their cardiovascular risk was increased, since the ratio CR1 and the At.C were significantly higher than the rest. Surprisingly, they tended to have a lower concentration of *Fas1* mRNA than those treated with VH or LC. As for the gene expression of the enzymes related to the lipolytic activity *Had* and that of the transporters *Cd36*, *Cpt1*α and *Fatp5*, they were similar among the groups exposed to L12. Similarly, the mRNA levels of the *Acc1* and *Srebp-1c* lipogenic enzymes showed no significant difference. It was also observed that animals treated with VH had significantly higher glycemia than those treated with cherries, regardless of origin. However, insulin levels among groups were similar. This could be due to the fiber content present in the administered lyophilizate, allowing a delay in gastric emptying and a decreased response to glycemic load [36,40]. Furthermore, the content of polyphenols, mainly anthocyanins, also exerts hypoglycemic effects. In this sense, there is evidence that the consumption of cherry juice inhibits the activity of the enzyme α-glucosidase and dipeptil-peptidase 4, improving glycemia [41]. 

The L6-nLC animals showed significantly higher gene expression of the hepatic lipogenic enzyme *Fas1*, which caused higher plasma TAG concentration than the rest of the treated animals. Surprisingly, when the two types of cherry were ingested out of season, only the animals that consumed the nLC showed this elevation of the TAG, while those fed with LC were similar to their VH. This situation could be showing that the nLC polyphenol phytochemical signature could be causing an alteration in the lipid metabolism when consumed in a season opposite to that of its harvest, which is not observed with the local fruit. It should be noted that, despite the fact that the harvest season of the fruits was the same, the transport conditions are crucial in the preservation of the polyphenolic content [22,42,43]. Specifically, temperature, relative humidity, exposure to light and transfer time are the factors that have the greatest influence on the useful life of bio-compounds. Cold storage could produce an increase in some phenolic compounds, while that of anthocyanidins could decrease by up to 52% in 15 days [44]. Actually, in our previous manuscript, we observed that LC had a significantly higher content of TPC, TAC, TFaC and TFoC than nLC [21]. In addition, cherry intake, regardless of its origin, significantly increased the circulating insulin levels compared to its respective VH. However, this situation did not affect the blood glucose of the animals. It should be noted that rats, being nocturnal animals, have more hours of movement in the short photoperiod. Therefore, it could be assumed that increased activity may be a secondary mechanism by which glucose is consumed; hence, it might be potentially responsible for the absence of statistically significant differences in their concentration between the different groups. On the other hand, it is important to note that the insulin levels observed in the present study are higher than those reported for similar experiences [45]. In this sense, this difference could be explained by the fact that the treatment dose or VH that entails a carbohydrate supply is carried out one hour before sacrifice. On the other hand, it was seen that the exposure to different photoperiods caused significant differences in the gene expression of lipogenic enzymes. Specifically, a clear effect was observed on the VH-treated groups, where exposure to L18 photoperiod significantly increased *Srebp-1c* and *Fas1* mRNA levels, compared to L12 or L6. It should be clarified that *Srebp-1c* improves the transcription of genes necessary for the synthesis of fatty acids, such as citrate lyase ATP, *Acc1* and *Fas1*, while limiting the gene expression of enzymes involved in the synthesis of TAG [46]. Therefore, this increased *Fas1* response can be expected in the L18-VH group, although no significant differences in *Acc1* expression were found among the VHs. Shimano et al. (1997) stated that an overexpression of hepatic *Srebp-1c* in transgenic mice increases up to four times the amount of mRNA of enzymes involved in the synthesis of fatty acids, including *Fas1*, while plasma TAG levels decrease [47]. The transcription of SREBP-1C is mainly regulated by three factors; LXRs and insulin promote its activity while glucagon inhibits it [46]. Although glucagon values and LXRs expression were not analyzed in the present study, plasma insulin values showed that the L18-VH group had the highest levels compared to the rest of the photoperiods. Therefore, a higher concentration of this anabolic hormone might be stimulating the expression of *Srebp-1c*, which, in turn, stimulates the expression of the *Acc1* and *Fas1*, despite this fact not being reflected in the actual concentration of plasma lipids. In this sense, there is evidence showing that mice with high levels of lipogenic enzymes can present a decrease in the rates of fatty acid synthesis, due to post-translational regulation through changes in phosphorylation and activation of the activity of key enzymes for the process [46].

By observing the effects of cherry consumption from different origins in the three photoperiods under study, we showed that the groups fed with cherry in L18 expressed more *Acc1* and *Fas1* mRNAs than the animals that consumed it out of season, either in L6 or in L12. However, this difference was only significant with LC. The precursor malonyl-CoA, synthesized by *Acc1*, causes the inhibition of *Cpt1α*, key gene for the oxidation of fatty acids in the mitochondria [48]. As expected, we could confirm this, showing that animals fed with LC in L18 had a lower expression of *Cpt1α* and, at the same time, that *Acc1* was increased with respect to the consumption of LC in L6. In this sense, it has been recently observed that animals exposed to L18, regardless of the one received, presented a higher % of body fat than those exposed to L6, which is how the higher expression of lipogenic enzymes would be explained [21]. Furthermore, although a lower expression of lipogenic enzymes was observed, it was not reflected in the concentration of plasma lipids, since the L6-nLC animals presented significantly higher levels of TAG than those that received nLC in L12 or in L18. Although this effect was only observed on TAG in L6-nLC, other authors have suggested that an increase in TAG in L6 in animals fed with STD could be due to a lower uptake of TAG by the white adipose tissue (WAT) than those exposed to L12 or L18 [27]. Although there is little evidence on the differential effects of consuming the same fruit but from different sources on metabolic parameters, Iglesias-Carres et al. observed that the phenolic composition of oranges from the southern and northern hemispheres were similar, although they differed in the type of compounds that predominated in each [49]. Furthermore, hepatic metabolism is regulated by the synchronization or desynchronization of the nuclear receptors related to clock genes, which are modulated not only by the circadian and circannual rhythm but also by the polyphenols and melatonin present in the fruits [20,25,26]. Likewise, the L6-nLC animals, in addition to presenting the highest levels of TAG, also had a higher concentration of plasma NEFAs than the L18-nLC group. A possible mechanism could be that the consumption of cherry in season, i.e., L18, stimulates a greater uptake of NEFAs by some fat deposit or oxidative tissue compared with L6. In this sense, there is evidence to suggest that fruits with a high content of polyphenols, such as grapes and cherries, increase lipid catabolism by increasing the absorption and oxidation of fatty acids in muscle compared to VH [25,50]. A higher concentration of circulating NEFAs is strongly associated with faulty insulin signaling [51]. These findings are consistent with our results, since animals that consumed cherry during the off-season showed higher insulin levels than those that ingested them in L18. Furthermore, both groups showed similar blood glucose levels, which may indicate that consuming cherry in L18 could improve insulin sensitivity. It should be noted that the plasma insulin levels in the groups treated with VH, nLC and LC in L18 show a completely opposite pattern to that observed in L6.

In the present study, we observed that there was an effect of the photoperiod and the treatment on the TC/HDL-c ratio and on the At.C. Due to the detrimental effect of a high content of circulating LDL-c and TAG and its correlation with very low-density lipoproteins (VLDL-c), the plasma concentrations of these lipids have been used for years as predictors of coronary heart disease. However, currently, there are other measures of dyslipidemia that are predictive for this type of pathology [52]. Specifically, a high TC/HDL-c ratio is positively related to obesity and early insulin resistance, being a more efficient parameter for diagnosing cardiovascular diseases obstructive and atherosclerotic plaque, even when normal LDL-c values are present [53]. Particularly, we observed that the animals fed with LC in L12 presented a TC/HDL-c and an At.C significantly higher than the animals belonging to the same photoperiod and even than those that ingested LC in L6 and L18. Similarly, this situation was repeated in the other photoperiods, where the LC group showed these elevated parameters in relation to the others, although they did not become significant. While it could be understood that off-season consumption of cherry causes undesirable effects on these atherogenic markers, providing erroneous signals according to the xenohormesis theory, it is not explained why, in L18, which resembled the growing season of the fruit, the LC produced this increase in the same way. However, the animals that consumed cherry within L18, presented the lowest AI values compared to their VH and the other groups, although the differences were not significant. It should be noted that higher AI is directly associated with high body weight, BMI, higher blood pressure levels and higher risk of insulin resistance [54]. In addition, it is also related to smaller and denser cholesterol particles, associated with higher long-term mortality in patients with cardiovascular risk factors [55]. Therefore, despite the fact that animals that consumed fruit in L18 had a TC/HDL-c and At.C higher than the other animals, their cardiovascular risk was offset by the protective effect that a lower AI gave them.

## 5. Conclusions

We report that there was a clear effect of exposure to different photoperiods on the plasma concentration of blood lipids and on the expression of liver lipogenic enzymes, while the treatment also tended to affect the glucidic metabolism and the gene expression of hepatic enzymes in Fischer 344 normal weight rats. We observed a significant effect of the photoperiod on the levels of TAGs and NEFAs, which modulated the values of the atherogenic index, the cardiovascular risk factor 1 and the atherogenic coefficient. On the other hand, the different treatments evaluated (VH, LC and nLC) significantly affected the cardiovascular risk factor 1 and the atherogenic coefficient. An interaction of both parameters (photoperiod and treatment) was observed in the levels of insulin and the HOMA index. We conclude that, considering the xenohormesis theory, the differential effect on the metabolic response could be affected by the origin and the time of consumption of this fruit, possibly due to the different content of bioactive compounds given by the polyphenolic signature of each of the cherries.

## Figures and Tables

**Table 1:** Plasma parameters and atherogenic indices in Fischer 344 rats exposed to different photoperiods and supplemented with local or non-local cherry or vehicle for 7 weeks.

	L6			L12			L18			
	nLC	LC	VH	nLC	LC	VH	nLC	LC	VH	*2wA*
**Serum parametrs**										
TAG (mmol/L)	1.42 ± 0.12 *a*	1.03 ± 0.14 *b*	1.14 ± 0.11 a *b	1.11 ± 0.08 *b*	1.11 ± 0.10 *b*	1.16 ± 0.08 a * b	1.00 ± 0.10 *b*	0.96 ± 0.10 *b*	0.95 ± 0.08 *b*	*P*
TC (mmol/L)	2.07 ± 0.22	1.94 ± 0.21	1.80 ± 0.16	2.21 ± 0.15	2.21 ± 0.14	1.97 ± 0.20	1.98± 0.23	1.84 ±0.21	1.97 ± 0.25	
HDL-c (mmol/L)	0.82 ± 0.17 *ab*	0.72 ± 0.17 *ab*	0.75 ± 0.11 *ab*	0.78 ± 0.17 *ab*	0.45 ± 0.08 ab *	0.65 ± 0.18 *ab*	0.89 ± 0.19 a *b	0.94 ± 0.29 *b*	0.38 ± 0.16 *a*	
LDL-c (mmol/L)	0.18 ± 0.05	0.20 ± 0.05	0.16 ± 0.06	0.16 ± 0.06	0.08 ± 0.02	0.09 ± 0.02	0.18 ± 0.06	0.21 ± 0.04	0.11 ± 0.03	
NEFAs (mmol/L)	0.79 ± 0.05 *a*	0.69 ± 0.09 *ab*	0.79 ± 0.10 *a*	0.79 ± 0.06 *a*	0.76 ± 0.07 *a*	0.78 ± 0.06 *a*	0.55 ± 0.05 *b*	0.56 ± 0.06 *b*	0.76 ± 0.08 *a*	*P*
Glucose (mmol/L)	8.69 ± 0.43 *ab*	8.46 ± 0.46 ab *	9.38 ± 0.69 *ab*	8.32 ± 0.92 *a*	8.06 ± 0.52 *a*	9.91 ± 0.55 *b*	8.24 ± 0.57 *a*	8.49 ± 0.19 ab *	8.50 ± 0.19 ab *	T *
Insulin (ng/mL)	9.86 ± 1.69 *a*	10.65 ± 1.56 *a*	5.37 ± 0.63 *b*	9.22 ± 1.71 *a*	9.07 ± 1.45 *ab*	8.95 ±1.65 ab *	8.12 ± 1.03 *ab*	7.07 ± 0.77 *ab*	9.14 ± 1.32 ab *	PxT *
HOMA	0.16 ± 0.03 *a*	0.17 ± 0.03 *a*	0.09 ± 0.01 *b*	0.15 ± 0.04 *ab*	0.13 ± 0.02 *ab*	0.18 ± 0.04 a	0.12 ± 0.02 *ab*	0.11 ± 0.01 *ab*	0.13 ± 0.01 *ab*	PxT *
**Atherogenic ratios**										
AI	0.24 ± 0.09	0.24 ± 0.12	0.14 ± 0.13	0.21 ± 0.09	0.44 ± 0.11 *	0.38 ± 0.13	0.03 ± 0.12	0.13 ± 0.13 *	0.33 ± 0.20	*P* *
CR1	2.13 ± 0.33 *a*	3.90 ± 0.86 *a*	1.97 ± 0.32 *a*	3.69 ± 0.62 *a*	6.38 ± 1.30 *b*	3.63 ± 0.51 *a*	2.00 ± 0.39 *a*	3.19 ± 0.74 *a*	2.80 ± 0.45 *a*	*P, T*
CR2	0.20 ± 0.05	0.34 ± 0.07	0.27 ± 0.13	0.30 ± 0.10	0.23 ± 0.06	0.10 ± 0.03	0.16 ± 0.05	0.30 ± 0.05	0.24 ± 0.12	
At.C	1.13 ± 0.33 *a*	2.90 ± 0.86 a	0.97 ± 0.32 *a*	2.69 ± 0.62 *a*	5.38 ± 1.30 *b*	2.63 ± 0.51 *a*	1.00 ± 0.39 *a*	2.19 ± 0.74 *a*	1.80 ± 0.45 *a*	*P, T*

Animals were exposed to a short, standard or long photoperiod, with 6 (L6), 12 (L12) or 18 (L18) hours of light, respectively, and were supplemented with vehicle (VH), with local cherry (LC) or non-local cherry (nLC). Data are expressed as the mean ± SEM (*n* = 8). Two-way ANOVA analysis (2 × 2 photoperiod factorial design (L6, L12 or L18) x treatment (VH, LC or nLC), was used to assess the differences between groups. P, photoperiod; T, treatment effect; PxT, effect of Interaction. TAG, triacylglyceride, TC, total cholesterol, HDL-c, high-density lipoprotein, LDL-c, low density lipoprotein, NEFAs, non-esterified fatty acids, HOMA, homeostatic model assessment, AI, atherogenic index (Log (TAG/HDL-c), CR1, cardiovascular risk 1 (TC/HDL-c), CR2, cardiovascular risk 2 (LDL-c/HDL-c), At.C, atherogenic coefficient ([TC-HDL-c]/HDL-c). Different letters above the bars indicate significant differences (*p* <0.05) (post-hoc DMS, one-way ANOVA). * Indicates trend (0.05 < *p* <1).

## Supplementary Materials

The following are available online at https://www.mdpi.com/article/10.3390/nu13103643/s1, Table S1: Nucleotide sequences of primers used for real time quantitative PCR.

## References

[1] Heimler D., Romani A., Ieri F. (2017). Plant polyphenol content, soil fertilization and agricultural management: A review. Eur. Food Res. Technol..

[2] Vita J.A. (2005). Polyphenols and cardiovascular disease: Effects on endothelial and platelet function. Am. J. Clin. Nutr..

[3] Fraga C.G., Oteiza P.I. (2011). Dietary flavonoids: Role of (-)-epicatechin and related procyanidins in cell signaling. Free Radic. Biol. Med..

[4] Oppedisano F., Muscoli C., Musolino V., Carresi C., Macrì R., Giancotta C., Bosco F., Maiuolo J., Scarano F., Paone S. (2020). The Protective Effect of Cynara Cardunculus Extract in Diet-Induced NAFLD: Involvement of OCTN1 and OCTN2 Transporter Subfamily. Nutrients.

[5] Subramanian G., Shanmugamprema D., Subramani R., Muthuswamy K., Ponnusamy V., Tankay K., Velusamy T., Krishnan V., Subramaniam S. (2021). Anti-Obesity Effect of T. Chebula Fruit Extract on High Fat Diet Induced Obese Mice: A Possible Alternative Therapy. Mol. Nutr. Food Res..

[6] Afrin S., Giampieri F., Gasparrini M., Forbes-Hernandez T.Y., Varela-López A., Quiles J.L., Mezzetti B., Battino M. (2016). Chemopreventive and therapeutic effects of edible berries: A focus on colon cancer prevention and treatment. Molecules.

[7] Sharma A., Kaur M., Katnoria J.K., Nagpal A.K. (2017). Polyphenols in Food: Cancer Prevention and Apoptosis Induction. Curr. Med. Chem..

[8] Martí R., Roselló S., Cebolla-Cornejo J. (2016). Tomato as a source of carotenoids and polyphenols targeted to cancer prevention. Cancers.

[9] Singla R.K., Dubey A.K., Garg A., Sharma R.K., Fiorino M., Ameen S.M., Haddad M.A., Al-Hiary M. (2019). Natural polyphenols: Chemical classification, definition of classes, subcategories, and structures. J. AOAC Int..

[10] Cubero J., Toribio F., Garrido M., Hernández M.T., Maynar J., Barriga C., Rodríguez A.B. (2010). Assays of the amino acid tryptophan in cherries by HPLC-fluorescence. Food Anal. Methods.

[11] Commisso M., Bianconi M., Di Carlo F., Poletti S., Bulgarini A., Munari F., Negri S., Stocchero M., Ceoldo S., Avesani L. (2017). Multi-approach metabolomics analysis and artificial simplified phytocomplexes reveal cultivar-dependent synergy between polyphenols and ascorbic acid in fruits of the sweet cherry (*Prunus avium* L.). PLoS ONE.

[12] McCune L.M., Kubota C., Stendell-Hollis N.R., Thomson C.A. (2011). Cherries and health: A review. Crit. Rev. Food Sci. Nutr..

[13] Connolly D.A.J., McHugh M.P., Padilla-Zakour O.I. (2006). Efficacy of a tart cherry juice blend in preventing the symptoms of muscle damage. Br. J. Sports Med..

[14] Kelley D.S., Adkins Y., Laugero K.D. (2018). A review of the health benefits of cherries. Nutrients.

[15] Xie X., Zhao B., Huang L., Shen Q., Ma L., Chen Y., Wu T., Fu Z. (2017). Effects of altered photoperiod on circadian clock and lipid metabolism in rats. Chronobiol. Int..

[16] Mattson M.P. (2008). Dietary factors, hormesis and health. Ageing Res. Rev..

[17] Hooper P.L., Hooper P.L., Tytell M., Vígh L. (2010). Xenohormesis: Health benefits from an eon of plant stress response evolution. Cell Stress Chaperones.

[18] Baur J.A., Sinclair D.A. (2008). What is xenohormesis?. Am. J. Pharmacol. Toxicol..

[19] Arola-Arnal A., Cruz-Carrión Á., Torres-Fuentes C., Ávila-Román J., Aragonès G., Mulero M., Bravo F.I., Muguerza B., Arola L., Suárez M. (2019). Chrononutrition and polyphenols: Roles and diseases. Nutrients.

[20] Mariné-Casadó R., Domenech-Coca C., del Bas J.M., Bladé C., Caimari A., Arola L. (2019). Cherry consumption out of season alters lipid and glucose homeostasis in normoweight and cafeteria-fed obese Fischer 344 rats. J. Nutr. Biochem..

[21] Cruz-Carrión Á., de Azua M.J.R., Mulero M., Arola-Arnal A., Suárez M. (2020). Oxidative stress in rats is modulated by seasonal consumption of sweet cherries from different geographical origins: Local vs. non-local. Nutrients.

[22] Ioannou I., Hafsa I., Hamdi S., Charbonnel C., Ghoul M. (2012). Review of the effects of food processing and formulation on flavonol and anthocyanin behaviour. J. Food Eng..

[23] Iglesias-Carres L., Mas-Capdevila A., Bravo F.I., Bladé C., Arola-Arnal A., Muguerza B. (2019). Optimization of extraction methods for characterization of phenolic compounds in apricot fruit (*Prunus armeniaca*). Food Funct..

[24] Iglesias-Carres L., Mas-Capdevila A., Isabel Bravo F., Mulero M., Muguerza B., Arola-Arnal A. (2019). optimization and characterization of Royal Dawn cherry (*Prunus avium*) phenolics extraction. Sci. Rep..

[25] Mariné-Casadó R., Domenech-Coca C., del Bas J.M., Bladé C., Arola L., Caimari A. (2018). The exposure to different photoperiods strongly modulates the glucose and lipid metabolisms of normoweight fischer 344 rats. Front. Physiol..

[26] Mariné-Casadó R., Domenech-Coca C., del Bas J.M., Bladé C., Arola L., Caimari A. (2018). Intake of an Obesogenic Cafeteria Diet Affects Body Weight, Feeding Behavior, and Glucose and Lipid Metabolism in a Photoperiod-Dependent Manner in F344 Rats. Front. Physiol..

[27] Gibert-Ramos A., Ibars M., Salvadó M.J., Crescenti A. (2019). Response to the photoperiod in the white and brown adipose tissues of Fischer 344 rats fed a standard or cafeteria diet. J. Nutr. Biochem..

[28] Sadowska J., Bruszkowska M. (2019). Assessing the effect of sugar type and form of its intake on selected parameters of carbohydrate-lipid metabolism and plasma atherogenic indices in rats. Rocz. Panstw. Zakl. Hig..

[29] Dobiášová M. (2017). Atherogenic impact of lecithin-cholesterol acyltransferase and its relation to cholesterol esterification rate in HDL (FERHDL) and AIP [log(TG/HDL-C)] biomarkers: The butterfly effect?. Physiol. Res..

[30] Sultani R., Tong D.C., Peverelle M., Lee Y.S., Baradi A., Wilson A.M. (2019). Elevated Triglycerides to High-Density Lipoprotein Cholesterol (TG/HDL-C) Ratio Predicts Long-Term Mortality in High-Risk Patients. Hear. Lung Circ..

[31] Rundek T., Blanton S.H., Bartels S., Dong C., Raval A., Demmer R.T., Cabral D., Elkind M.S.V., Sacco R.L., Desvarieux M. (2013). Traditional risk factors are not major contributors to the variance in carotid intima-media thickness. Stroke.

[32] Pfaffl M.W. (2004). Quantification strategies in real-time PCR. AZ Quant. PCR.

[33] Kassi E., Pervanidou P., Kaltsas G., Chrousos G. (2011). Metabolic syndrome: Definitions and controversies. BMC Med..

[34] Reagan-Shaw S., Nihal M., Ahmad N. (2008). Dose translation from animal to human studies revisited. FASEB J..

[35] Rui L. (2016). Energy Metabolism in the Liver. Physiol. Behav..

[36] Morgan A.E., Mooney K.M., Wilkinson S.J., Pickles N.A., Mc Auley M.T. (2016). Cholesterol metabolism: A review of how ageing disrupts the biological mechanisms responsible for its regulation. Ageing Res. Rev..

[37] Jia Y., Hoang M.H., Jun H.J., Lee J.H., Lee S.J. (2013). Cyanidin, a natural flavonoid, is an agonistic ligand for liver X receptor alpha and beta and reduces cellular lipid accumulation in macrophages and hepatocytes. Bioorganic Med. Chem. Lett..

[38] Xia M., Hou M., Zhu H., Ma J., Tang Z., Wang Q., Li Y., Chi D., Yu X., Zhao T. (2005). Anthocyanins induce cholesterol efflux from mouse peritoneal macrophages: The role of the peroxisome proliferator-activated receptor γ-liver X receptor α-ABCA1 pathway. J. Biol. Chem..

[39] Wang D., Xia M., Yan X., Li D., Wang L., Xu Y., Jin T., Ling W. (2012). Gut microbiota metabolism of anthocyanin promotes reverse cholesterol transport in mice via repressing miRNA-10b. Circ. Res..

[40] Dikeman C.L., Fahey G.C. (2006). Viscosity as related to dietary fiber: A review. Crit. Rev. Food Sci. Nutr..

[41] Cásedas G., Les F., Gómez-Serranillos M.P., Smith C., López V. (2016). Bioactive and functional properties of sour cherry juice (*Prunus cerasus*). Food Funct..

[42] Remón S., Venturini M.E., Lopez-Buesa P., Oria R. (2003). Burlat cherry quality after long range transport: Optimisation of packaging conditions. Innov. Food Sci. Emerg. Technol..

[43] Correia S., Schouten R., Silva A.P., Gonçalves B. (2017). Factors affecting quality and health promoting compounds during growth and postharvest life of sweet cherry (*Prunus avium* L.). Front. Plant Sci..

[44] Esti M., Cinquanta L., Sinesio F., Moneta E., Di Matteo M. (2002). Physicochemical and sensory fruit characteristics of two sweet cherry cultivars after cool storage. Food Chem..

[45] Gibert-Ramos A., Palacios-Jordan H., Salvadó M.J., Crescenti A. (2020). Consumption of out-of-season orange modulates fat accumulation, morphology and gene expression in the adipose tissue of Fischer 344 rats. Eur. J. Nutr..

[46] Horton J.D., Goldstein J.L., Brown M.S. (2002). SREBPs. Most.

[47] Shimano H., Horton J.D., Shimomura I., Hammer R.E., Brown M.S., Goldstein J.L. (1997). Isoform 1c of sterol regulatory element binding protein is less active than isoform 1a in livers of transgenic mice and in cultured cells. J. Clin. Investig..

[48] Abu-Elheiga L., Brinkley W.R., Zhong L., Chirala S.S., Woldegiorgis G., Wakil S.J. (2000). The subcellular localization of acetyl-CoA carboxylase 2. Proc. Natl. Acad. Sci. USA.

[49] Iglesias-Carres L., Mas-Capdevila A., Bravo F.I., Aragonès G., Muguerza B., Arola-Arnal A. (2019). Optimization of a polyphenol extraction method for sweet orange pulp (*Citrus sinensis* L.) to identify phenolic compounds consumed from sweet oranges. PLoS ONE.

[50] Crescenti A., del Bas J.M., Arola-Arnal A., Oms-Oliu G., Arola L., Caimari A. (2015). Grape seed procyanidins administered at physiological doses to rats during pregnancy and lactation promote lipid oxidation and up-regulate AMPK in the muscle of male offspring in adulthood. J. Nutr. Biochem..

[51] Samuel V.T., Shulman G.I. (2016). The pathogenesis of insulin resistance: Integrating signaling pathways and substrate flux. J. Clin. Investig..

[52] Nair D., Carrigan T.P., Curtin R.J., Popovic Z.B., Kuzmiak S., Schoenhagen P., Flamm S.D., Desai M.Y. (2009). Association of total cholesterol/high-density lipoprotein cholesterol ratio with proximal coronary atherosclerosis detected by multislice computed tomography. Prev. Cardiol..

[53] Ozturk M.A. (2019). Association between cardiovascular risk factors and triglyceride to high-density lipoprotein ratio: A single-center experience. Arch. Med. Sci.-Atheroscler. Dis..

[54] Pantoja-Torres B., Toro-Huamanchumo C.J., Urrunaga-Pastor D., Guarnizo-Poma M., Lazaro-Alcantara H., Paico-Palacios S., del Carmen Ranilla-Seguin V., Benites-Zapata V.A. (2019). High triglycerides to HDL-cholesterol ratio is associated with insulin resistance in normal-weight healthy adults. Diabetes Metab. Syndr. Clin. Res. Rev..

[55] Endothelial Permeability, LDL Deposition, and Cardiovascular Risk Factors-A Review—PubMed. https://pubmed.ncbi.nlm.nih.gov/29228169-endothelial-permeability-ldl-deposition-and-cardiovascular-risk-factors-a-review/?from_term=LDL+and+risk+cardiovascular&from_pos=1.

