# Supplementary material for: Seasonal Consumption of Cherries from Different Origins Affects Metabolic Markers and Gene Expression of Lipogenic Enzymes in Rat Liver: A Preliminary Study
Source: Nutrients. 2021 Oct 18;13(10):3643. doi: 10.3390/nu13103643 (PMC8537345; doi:10.3390/nu13103643)
Supplement: Supplementary file 1 [file nutrients-13-03643-s001.zip › nutrients-1400625-supplementary.pdf]

Supplementary Material

Supplementary Table S1. Nucleotide sequences of primers used for real time quantitative PCR.

Gene	Forward Primer (5′ to 3′)	Reverse Primer (5′ to 3′)
Acc1	TGCAGGTATCCCCACTCTTC	TTCTGATTCCCTTCCCTCCT
Cd36	GTCCTGGCTGTGTTTGGA	GCTCAAAGATGGCTCCATTG
Cpt1α	GCTCGCACATTACAAGGACAT	TGGACACCACATAGAGGCAG
Fas1	CTATTGTGGACGGAGGTATC	TGCTGTAGCCCAGAAGAG
Fatp5	CCTGCCAAGCTTCGTGCTAAT	GCTCATGTGATAGGATGGCTGG
Had	ATCGTGAACCGTCTCTTGGT	AGGACTGGGCTGAAATAAGG
Srebp-1c	CCCACCCCCTTACACACC	GCCTGCGGTCTTCATTGT

The table shows the nucleotide sequences of primers used for PCR amplification. *Acc1*, acetyl CoA carboxylase; *Cd36*, fatty acid translocase, homologue of CD36; Cpt1*α*, carnitine palmitoyltransferase 1 alpha; Fas1, sterol regulatory element-binding protein 1; *Fatp5*, fatty acid transport protein 5; *Had*, hydroxyacyl-CoA dehydrogenase; *Srebp1c*, sterol regulatory element-binding protein 1c.
